# Subclinical Hyperthyroidism: Status of the Cholesterol Transfers to HDL and Other Parameters Related to Lipoprotein Metabolism in Patients Submitted to Thyroidectomy for Thyroid Cancer

**DOI:** 10.3389/fendo.2020.00176

**Published:** 2020-04-02

**Authors:** Gilbert A. Sigal, Thauany M. Tavoni, Bruna M. O. Silva, Roberto Khalil-Filho, Lenine G. Brandão, Edmund C. Baracat, Raul C. Maranhão

**Affiliations:** ^1^Lipid Metabolism Laboratory, Heart Institute (InCor) of the Medical School Hospital, University of São Paulo, São Paulo, Brazil; ^2^Clinical Cardiology Division, Heart Institute (InCor) of the Medical School Hospital, University of São Paulo, São Paulo, Brazil; ^3^Department of Head and Neck Surgery of the Medical School Hospital, University of São Paulo, São Paulo, Brazil; ^4^Department of Obstetrics and Gynecology, of the Medical School Hospital, University of São Paulo, São Paulo, Brazil; ^5^Faculty of Pharmaceutical Science, University of São Paulo, São Paulo, Brazil

**Keywords:** subclinical hyperthyroidism, lipoproteins, HDL function, cholesterol ester transfer protein, lecithin-cholesterol acyltransferase, LCAT, paraoxonase, PON-1

## Abstract

**Purpose:** Lipid metabolism has been poorly explored in subclinical hyperthyroidism. The aim was to examine the effects of exogenous subclinical hyperthyroidism in women under levothyroxine treatment upon plasma lipids and aspects of HDL metabolism.

**Methodology:** Ten women were studied in euthyroidism and again in exogenous subclinical hyperthyroidism. Thyroid function tests and plasma lipids were studied.

**Results:** HDL-cholesterol (increased 21.6%, *p* = 0.0004), unesterified cholesterol (increased 12.3%, *p* = 0.04) and Lp(a) (increased 33,3%, *P* = 0.02) plasma concentrations were higher in subclinical hyperthyroidism compared to euthyroidism, but total cholesterol, LDL, non-HDL cholesterol, triglycerides, apo A-I, apo B were unchanged. PON1 activity (decreased 75%, *p* = 0.0006) was lower in subclinical hyperthyroidism. There were no changes in HDL particle size, CETP and LCAT concentrations. The *in vitro* assay that estimates the lipid transfers to HDL showed that esterified cholesterol (increased 7.1%, *p* = 0.03), unesterified cholesterol (increased 7.8%, *p* = 0.02) and triglycerides (increased 6.8%, *p* = 0.006) transfers were higher in subclinical hyperthyroidism. There were no changes in phospholipid transfers to HDL in subclinical hyperthyroidism.

**Conclusions:** Several alterations in the plasma lipid metabolism were observed in the subclinical hyperthyroidism state that highlight the importance of this aspect in the follow-up of those patients. The increase in HDL-C and in the transfer of unesterified and esterified cholesterol to HDL, an important anti-atherogenic HDL function are consistently protective for cardiovascular health. The increase in Lp(a) and the decrease in PON-1 activity that are important risk factors were documented here in subclinical hyperthyroidism and these results should be confirmed in larger studies due to great data variation but should not be neglected in the follow-up of those patients.

## Introduction

Patients with differentiated thyroid cancer submitted to total thyroidectomy are thereafter maintained for prolonged time in exogenous subclinical hyperthyroidism state by administration of levothyroxine (LT4) at supraphysiological dose regimen. This standard protocol is aimed to prevent tumor relapse and in many of those patients the subclinical hyperthyroidism condition is sustained for life. However, when patients are in the subclinical hyperthyroidism state, defined as low TSH and normal free thyroxine serum concentration, there is increase in the risk of cardiovascular diseases such as atrial fibrillation (1, 2). The plasma lipid metabolism is strongly influenced by the action of the thyroid hormones (3) so that it is noticeable that the status of this metabolism in patients with induced subclinical hyperthyroidism has been poorly explored (4). In respect to endogenous subclinical hyperthyroidism, studies on the lipid status are also scarce and, moreover, controversial. PARLE et al. found no differences in LDL-C or HDL-C of subclinical hyperthyroidism patients and controls (5), differently from Bergouth et al. (6) that reported that LDL-C, HDL-C, and triglycerides were lower in this condition. Lipoprotein (a), [Lp(a)], that has been consistently recognized as risk factor has remained little explored in subclinical hyperthyroidism.

In regard to the HDL fraction, this lipoprotein has several functions related with anti-atherogenesis protection such as that in reverse cholesterol transport and in the esterification of cholesterol. HDL has also antioxidative and many other protective actions (7). In this context, HDL-C determination alone falls short from the understanding of the whole potential protective role of HDL.

Plasma lipids, such as unesterified and esterified cholesterol, triglycerides, and phospholipids are continuously exchanged among the lipoprotein classes. These lipid transfers are facilitated by the action of the transfer proteins cholesterol ester transfer protein (CETP) and phospholipid transfer protein (PLTP) and are important for the intravascular formation and metabolism of HDL and for the HDL function in cholesterol esterification and reverse cholesterol transport. Previously, we showed by an *in vitro* assay that the transfers of unesterified and esterified cholesterol were diminished in patients with coronary artery disease (8). We also showed that the transfers of phospholipids and triglycerides to HDL were diminished in patients in subclinical hypothyroidism (9), whereas in overt hypothyroidism the transfers of all those four lipids were diminished (10).

Since patients submitted to total thyroidectomy for thyroid cancer are often maintained for long periods in subclinical hyperthyroidism, with largely ignored alterations in plasma lipid metabolism, this study was aimed to investigate these metabolic aspects that can also pertain to endogenous subclinical hyperthyroidism.

## Materials and Methods

### Patients

Eighteen women that were being submitted to total thyroidectomy for differentiated thyroid cancer at the Department of Head and Neck Surgery of the Medical School Hospital of the University of São Paulo were enrolled in the study. In this standard post-surgery protocol, patients are maintained in hypothyroidism for 3 weeks in order to perform whole body scan for detection of residual thyroid tissue and possibly existing metastatic sites. In the ensuing period, patients are maintained in subclinical hyperthyroidism by continuous administration of levothyroxine. However, only 10 out of the 18 initially enrolled patients were selected for the study on the basis of their having achieved subclinical hyperthyroidism maintained for at least 5 months. They were aged 33–63 years (47 ± 9), BMI 28.6 ± 4.2 kg/m^2^. The remaining eight patients were excluded since subclinical hyperthyroidism was not documented when blood was sampled. The results of the total 18 women while in the hypothyroid state period were reported elsewhere (10).

All the procedures in this study were in accordance with the guidelines of the Helsinki Declaration on human experimentation. The study protocol was approved by the Ethical Committee of the Medical Hospital of the University of São Paulo, and written informed consent was obtained from all participants.

Blood was collected twice for biochemical analysis from all participants. The first blood withdrawal was made when they were in euthyroid state, just before they entered the surgery room to undergo thyroidectomy. The second blood sampling was taken when they were in use of LT4 (88 to 150 mcg/day). At this time they were for at least 5 months in the subclinical hyperthyroid state. Patients did not report changes in eating habits or physical activity between the first and second blood sampling.

After at least 12 h fast blood was collected between 8 a.m. and 14 p.m. and TSH, free T4, C-reactive protein (CRP), PON 1 activity, HDL size, cholesterol ester transfer protein(CETP), lecithin cholesterol acyl transferase(LCAT), total cholesterol, LDL-C, non-HDL cholesterol (non-HDL-C), HDL-C, triglycerides, apo A-I, apo-B, Lp(a) and unesterified cholesterol were determinated. An assay of *in vitro* lipid transfers to HDL was also determined.

### Serum Biochemical Determinations

Commercial enzymatic methods were used to determine total cholesterol (Boehringer-Mannheim, Penzberg, Germany), free cholesterol (Wako, Osaka, Japan) and triglycerides (Abbott, North Chicago, IL). HDL-C was measured by the same method used for total cholesterol after lipoprotein precipitation with magnesium phosphotungstate. LDL-C was calculated by the Friedewald Equation (11) and non-HDL-C was determined by the equation: total cholesterol minus HDL-C. Apolipoprotein (apo) A-I and apo B were measured by rate nephelometry on an Image Immunochemistry System (Beckman Coulter, Brea, CA). Lipoprotein (a) was determined by immunonephelometry (equipament BN II) commercial kit reagent N Latex Lp(a) (Siemens Healthcare Diagnostics Products GmbH. Serum TSH and free T4 were assayed by fluoroimmunoassay (AutoDELFIA equipment, AutoDELFIA Ultrakit, Wallac Oy) 4.4 and 3.4% for free T4, respectively. Serum C-reactive protein (CRP) was determinate by immunonephelometry method(BN II Systems, CARDIO PHASE® hs CRP, Siemens Healthcare, Marburg, Germany). The quantitative determination of CETP and LCAT in serum was determined by two different ELISA immunoassays (ALPCO Diagnostics, Salem, MA). PON1 activity was measured by adding serum to 1M Tris-HCl buffer (100 mmol/L, pH 8.0) containing 2 mmol/L CaCl_2_ and 5.5 mmol/L paraoxon (Sigma, Seelze, Germany). The generation of p-nitrophenol was measured at 405 nm, at 37°C in a plate reader (Victor™ X3, Perkin Elmer, Singapore) (12). The diameters of HDL particles were determined by Zetasizer nano ZS90 (Malvern, Worcestershire, UK) as described elsewhere (13). The *in vitro* assay of the simultaneous transfer of radioactively labeled phospholipids, triglycerides, unesterified, and esterified cholesterol from an artificial nanoparticle to the HDL plasma fraction was performed as described by Lo Prete et al. (14). In brief, the donor lipidic nanoparticle containing the labeled lipids was incubated during 1 h with whole plasma. After chemical precipitation of the nanoparticle and the apo B-containing lipoprotein fractions, the supernatant containing the HDL fraction was counted for radioactivity in a scintillation solution, and the percentage radioactivity of each lipid that transferred from the nanoparticle to HDL was then estimated. The HDL fraction was obtained from the whole plasma after precipitation of the apo B-containing lipoproteins with magnesium phosphotungstate. Triglyceride (Labtest, Minas Gerais, Brazil), unesterified cholesterol and phospholipid (Wako, Richmond, VA) were determined by using commercial kits. Esterified cholesterol was calculated as the difference between total and unesterified cholesterol of the HDL multiplied by 1.67 to adjust for M.W. of esterified cholesterol (15).

### Statistical Analysis

Results were presented as means ± standard deviation. Differences in results between euthyroidism phase and subclinical hyperthyroidism phase were assessed by Student's *t*-test. *p*-values < 0.05 were considered statistically significant. Statistical power of the test was calculated for the variables that have reached statistical significance.

## Results

As shown in Table 1, the euthyroid and subclinical hyperthyroidism states were confirmed in all patients by the values of TSH (1.6 ± 0.8 vs. 0.09 ± 0.13 uUI/mL, *p* = 0.0004) and free T4 (1.02 ± 0.16 vs. 1.44 ± 0.16 ng/dL, *p* < 0.0001).

**Table 1:** Thyroid and lipid metabolism parameters in euthyroidism and subclinical hyperthyroidism.

	**Euthyroidism (*n* = 10)**	**Hyperthyroidism (*n* = 10)**	***P*-value**
TSH (uUI/mL)	1.6 ± 0.8	0.09 ± 0.13	0.0004
Free T4 (ng/dL)	1.02 ± 0.16	1.44 ± 0.16	<0.0001
CRP (mg/L)	3.73 ± 5.77	3.20 ± 5.68	0.7137
Cholesterol (mg/dL)			
Total	190 ± 36	210 ± 36	0.0598
LDL	125 ± 35	134 ± 31	0.3405
HDL	37 ± 8	45 ± 9	0.0004
Non-HDL	153 ± 34	165 ± 33	0.2511
Unesterified Cholesterol (mg/dL)	57 ± 9	64 ± 9	0.0466
Triglycerides (mg/dL)	139 ± 39	170 ± 71	0.2211
Apolipoprotein (g/L)			
A-I	1.34 ± 0.22	1.39 ± 0.17	0.4634
B	1.00 ± 0.23	1.06 ± 0.20	0.3906
Lp(a) (mg/dL)	30 ± 23	40 ± 32	0.0179
CETP (ug/mL)	2.6 ± 1.4	2.7 ± 0.6	0.7792
LCAT (ug/mL)	8.1 ± 1.6	7.8 ± 1.9	0.7430
PON1 (U/L)	112 ± 52	64 ± 31	0.0006
HDL diameter (nm)	8.7 ± 0.4	9.1 ± 0.4	0.1027

*Data are expressed as mean , ±SD. TSH, thyroid-stimulating hormone; CRP, C-reactive protein; LDL, low density lipoprotein; HDL, high density lipoprotein; Lp(a), lipoprotein (a); CETP, cholesterol ester transfer protein; LCAT, lecithin-cholesterol acyltransferase; PON1, paraoxonase 1*.

**Figure 1:**
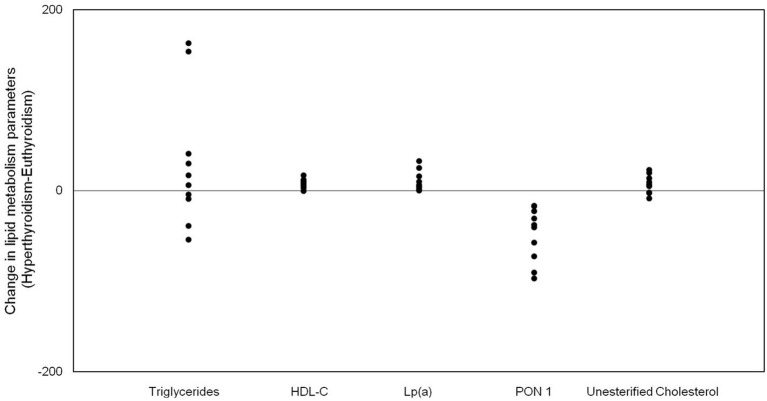
Scattered dot diagram of changes in lipid metabloism parameters between subclinical hyperthyroid and euthyroid state.

**Figure 2:**
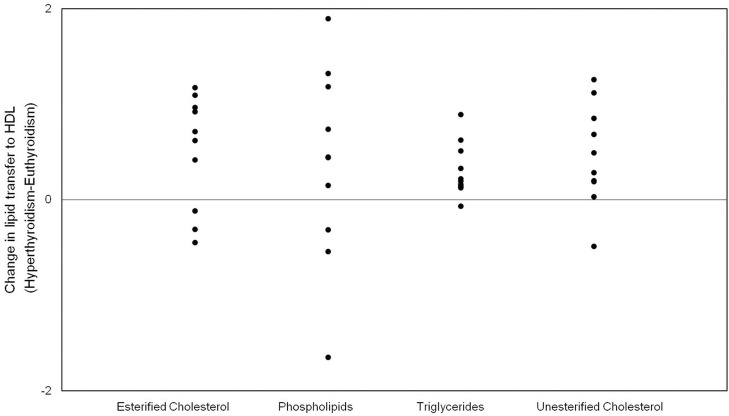
Scattered dot diagram of changes in lipid transfers to HDL between subclinical hyperthyroid and euthyroid state.

In subclinical hyperthyroidism, baseline serum levels of HDL-C (power: 99.7%), unesterified cholesterol (power: 52.9%) and Lp(a) (power: 73.0%) were elevated (Figure 1). The serum concentration of total cholesterol, LDL-C, non-HDL-C, triglycerides, apoA-1 and apoB were unchanged in subclinical hyperthyroidism. Table 1 also shows that the activity of PON-1 (power: 99.5%) was decreased in subclinical hyperthyroidism when compared to euthyroid state (Figure 1). The serum concentration of CETP and LCAT and the HDL particles diameter were equal in euthyroidism and subclinical hyperthyroidism Table 2 shows the % of transfer of the four radioactively labeled lipids, namely unesterified and esterified cholesterol, phospholipids, and triglycerides, from the artificial donor nanoparticle to the HDL fraction after 1 h incubation of the nanoemulsion with whole plasma. The transfers of esterified (power: 66.2%) and unesterified cholesterol (power:69.1%) and triglycerides (power: 88.5%) were higher in subclinical hyperthyroidism than in euthyroidism (Figure 2). Transfers of phospholipids did not change from the euthyroid to the subclinical hyperthyroid state (Figure 2).

**Table 2:** Lipid transfers to HDL in euthyroidism and subclinical hyperthyroidism.

**Lipid transfer (%)**	**Euthyroidism (*n* = 10)**	**Hyperthyroidism (*n* = 10)**	***P*-value**
Esterified cholesterol	6.99 ± 0.97	7.49 ± 0.90	0.0262
Unesterified Cholesterol	5.87 ± 1.24	6.33 ± 1.27	0.0220
Phospholipids	18.33 ± 1.47	17.61 ± 3.72	0.5071
Triglycerides	5.16 ± 0.83	5.51 ± 0.83	0.0063

*Data are expressed as mean , ±SD*.

## Discussion

The state of subclinical hyperthyroidism after the minimal 5-month period of TL4 treatment (14 ± 6 months) was confirmed in all participant patients by their low TSH and normal free T4 concentration. Subclinical hyperthyroidism neither changed their LDL-C nor the levels of apo B, the protein marker of the LDL fraction. Non-HDL-C and triglycerides were also unaffected when in the subclinical hyperthyroid state. For comparative ends, data in the literature on the effects of exogenous subclinical hyperthyroidism on plasma lipids are lacking and even data from endogenous subclinical hyperthyroidism are rather scanty.

Parle et al. (5) reported that in 27 aged patients in subclinical hyperthyroidism LDL-C was equal to those of controls, but triglyceride levels were not measured. Bergouth et al. (6) reported that in 11 multinodular goiter patients with subclinical hyperthyroidism LDL-C and triglycerides were lower than in 15 euthyroid controls. Parle et al. found that HDL-C was not different from the controls in their subclinical hyperthyroid patients, whereas Bergouth found that HDL-C was lower in theirs. The increased HDL-C in our patients when in the subclinical hyperthyroid state was not accompanied by a significantly increased apo A-I, the main apo found in the HDL fraction and also a marker of the HDL level.

It is worth while to point out that in overt hyperthyroidism, in which the plasma lipids have been well-documented, both pro-atherogenic LDL and anti-atherogenic HDL levels are diminished, differently from the effects of subclinical hyperthyroidism observed in the current study. Triglycerides are not consistently altered in overt hyperthyroidism, and were found increased (16, 17), decreased or unchanged (17).

Lp(a) is increasingly recognized as a CAD risk factor and high Lp(a) plasma levels have also been related with the incidence of aortic valve calcification and stenosis (18). Until recently, there were no effective therapeutic tools to lower Lp(a), but the introduction of the PCSK9 inhibitors has fulfilled this gap and the effects of lowering Lp(a) will probably be soon evaluated. Reports on Lp(a) status in overt hyperthyroid are scanty. De Bruin et al. (19) found that Lp(a) was lower in 27 patients with overt hyperthyroidism compared to their euthyroid controls, but it is important to point out that Lp(a) variation spectrum in the plasma is fairly wide, which can eventually mislead the results when comparing small groups of subjects. At any rate, that study was confirmed by Bonde et al. (20) that treated 20 hyperthyroid patients and found that after they reached the euthyroid state Lp(a) increased.

There are few information about the status of Lp(a) in endogenous or exogenous subclinical hyperthyroidism and our finding of 33% increase in Lp(a) levels in our patients is noteworthy. Thyroid autoimmunity was shown to increase Lp(a) regardless of the thyroid function status (21), but in our study the existence of thyroid autoimmunity was discarded, since our patients had been submitted to total thyroidectomy and radioiodine therapy, with absence or near-absence of thyroid tissue confirmed by the very low plasma thyroglobulin levels. It is interesting to point out that CRP was unchanged from the euthyroid state to subclinical hyperthyroidism, which suggests that this increase in Lp(a) was not related with inflammation-based changes. In this setting, it is difficult to identify factors whereby the acquisition of subclinical hyperthyroidism was accompanied by higher Lp(a).

In this study, we found that there was increase in the rates of transfer to the HDL fraction of unesterified and esterified cholesterol and triglycerides; the phospholipid transfer was unchanged. Increased transfers were conceivably due to the ≅21% increase in HDL-C since HDL concentration is an influent factor for the transfers and CETP, that stimulates these transfers, was unchanged in the subclinical hyperthyroid state. The HDL fraction, to which both LCAT and its co-factor apo A-I are associated, is the preferential site of plasma cholesterol esterification (22). Thus, it is somewhat unexpected that the plasma unesterified cholesterol concentration was augmented in the subclinical hyperthyroid state, since both HDL-C and the unesterified cholesterol transfer to HDL were increased. It is interesting to compare our current lipid transfer results on subclinical hyperthyroidism with those from our previous studies on subclinical hypothyroidism (9) and over hypothyroidism (10). In subclinical hypothyroidism, there was a decrease in the transfer of phospholipids and triglycerides to HDL while in overt hypothyroidism we observed a decrease of transfer of phospholipids, triglycerides, esterified and unesterified cholesterol to HDL after adjusted by HDL-C. Since deficiency and excess of thyroid hormone have opposite effects on metabolism, it is crucial to emphasize that the overall results of the lipid transfers to HDL have been consistent with the thyroid functional status: while overt and subclinical hypothyroidism led to decreased lipid transfer, subclinical hyperthyroidism was, in contrast, followed by increased lipid transfers.

PON-1, an enzyme associated to the HDL fraction has anti-oxidative action, specially upon the LDL fraction. PON-1 promotes the hydrolysis of oxidized phospholipids. In the current study, the subclinical hyperthyroid state lead to a marked reduction of the PON-1 activity. This effect may have important repercussions on the protective anti-atherosclerosis effects of the enzyme. Reduced PON-1 activity was also found in patients with multinodular goiter kept in subclinical hyperthyroidism by treatment with suppressive doses of LT4 (23). It is of interest to point out that the overall novel alterations found in this study may eventually be present in subclinical hyperthyroid states of other etiologies.

The small number of cases studied previous and after the subclinical hyperthyroid state consists in a limitation of the study, due to the well-known large variations of Lp(a) and PON-1 values. To our knowledge PON-1 and Lp(a) were scarcely explored in subclinical hyperthyroidism and the alterations found here should be approached in further studies enrolling larger number of patients, due to the great epidemiologic impact of Lp(a) and PON-1 as risk factors for atherosclerotic cardiovascular diseases.

## Conclusion

In conclusion, the increase in both unesterified and esterified cholesterol transfer to HDL in subclinical hyperthyroidism found here can be an important anti-atherosclerosis mechanism in this condition, as indicated in previous studies. The opposite effects of subclinical hyperthyroidism on one hand and of subclinical and overt hypothyroidism on the other reinforce the importance of the thyroid function on the cholesterol fluxes among lipoproteins in the plasma. The changes in Lp(a) concentration and PON-1 activity need larger studies for confirmation.

## Data Availability Statement

The datasets generated for this study are available on request to the corresponding author.

## Ethics Statement

The studies involving human participants were reviewed and approved by Ethical Committee of the Medical Hospital of the University of São Paulo. The patients/participants provided their written informed consent to participate in this study.

## Author Contributions

GS: conception and design of the study, patient selection, collecting clinical information, data collection and analysis, and drafting the manuscript. TT: data collection and analysis, performance of experiments, and statistical analysis. BS: data collection and performance of experiments. RK-F: critical revision. EB: critical revision. LB: conception and design of the study and critical revision. RM: conception, design, and conduction of the study, interpretation of data, and manuscript writing. All authors have revised and approved the submitted manuscript.

### Conflict of Interest

The authors declare that the research was conducted in the absence of any commercial or financial relationships that could be construed as a potential conflict of interest.
